# Machine Learning-Powered Smart Healthcare Systems in the Era of Big Data: Applications, Diagnostic Insights, Challenges, and Ethical Implications

**DOI:** 10.3390/diagnostics15151914

**Published:** 2025-07-30

**Authors:** Sita Rani, Raman Kumar, B. S. Panda, Rajender Kumar, Nafaa Farhan Muften, Mayada Ahmed Abass, Jasmina Lozanović

**Affiliations:** 1 Department of Computer Science and Engineering, Guru Nanak Dev Engineering College, Ludhiana 141006, Punjab, India; sitasaini80@gmail.com; 2 Department of Mechanical and Production Engineering, Guru Nanak Dev Engineering College, Ludhiana 141006, Punjab, India; 3 Jadara Research Center, Jadara University, Irbid 21110, Jordan; 4 Department of CSE, Raghu Engineering College, Visakhapatnam 531162, Andhra Pradesh, India; panda.bs@raghuenggcollege.in; 5 Department of Mechanical Engineering, Graphic Era (Deemed to be University), Clement Town, Dehradun 248002, Uttarakhand, India; rajender6291@gmail.com; 6 Centre for Research Impact & Outcome, Chitkara University Institute of Engineering and Technology, Chitkara University, Rajpura 140401, Punjab, India; 7 Department of Medical Laboratories Technology, Mazaya University College, Nasiriyah 64001, Iraq; nafaaalomari10@gmail.com; 8 College of Pharmacy, Al-Mustaqbal University, Babylon 51001, Iraq; mayada.ahmed@uomus.edu.iq; 9 Department of Engineering, FH Campus Wien, University of Applied Sciences, Favoritenstraße 226, 1100 Vienna, Austria

**Keywords:** smart healthcare systems, smart diagnostics, artificial intelligence, machine learning, big data, electronic health records (EHRs)

## Abstract

Healthcare data rapidly increases, and patients seek customized, effective healthcare services. Big data and machine learning (ML) enabled smart healthcare systems hold revolutionary potential. Unlike previous reviews that separately address AI or big data, this work synthesizes their convergence through real-world case studies, cross-domain ML applications, and a critical discussion on ethical integration in smart diagnostics. The review focuses on the role of big data analysis and ML towards better diagnosis, improved efficiency of operations, and individualized care for patients. It explores the principal challenges of data heterogeneity, privacy, computational complexity, and advanced methods such as federated learning (FL) and edge computing. Applications in real-world settings, such as disease prediction, medical imaging, drug discovery, and remote monitoring, illustrate how ML methods, such as deep learning (DL) and natural language processing (NLP), enhance clinical decision-making. A comparison of ML models highlights their value in dealing with large and heterogeneous healthcare datasets. In addition, the use of nascent technologies such as wearables and Internet of Medical Things (IoMT) is examined for their role in supporting real-time data-driven delivery of healthcare. The paper emphasizes the pragmatic application of intelligent systems by highlighting case studies that reflect up to 95% diagnostic accuracy and cost savings. The review ends with future directions that seek to develop scalable, ethical, and interpretable AI-powered healthcare systems. It bridges the gap between ML algorithms and smart diagnostics, offering critical perspectives for clinicians, data scientists, and policymakers.

## 1. Introduction

The rapid advancement of digital technologies and the integration of networked biomedical devices have profoundly transformed the healthcare landscape, resulting in a substantial increase in the volume, variety, and complexity of healthcare data [1]. Intelligent health systems that leverage machine learning (ML) and big data analytics (BDA) are poised to revolutionize care delivery by enabling advanced prediction, personalized treatment, and real-time patient monitoring [2]. Despite this potential, several critical challenges remain. These include heterogeneity in data sources, ranging from wearable sensors and genomic data to electronic health records (EHRs), along with growing concerns around data privacy in an increasingly hostile cyber threat environment, the computational burden of real-time analytics, and the interpretability and clinical validity of ML-driven decision support systems [3]. Addressing these challenges necessitates the development of scalable, workflow-compatible, and regulatory-compliant solutions, thereby motivating the focus of this review.

The healthcare sector is already experiencing an exponential growth in data volume, primarily attributed to the proliferation of EHR systems, wearable devices, and Internet of Things (IoT) enabled medical technologies. As of 2023, the global healthcare big data market was valued at approximately USD 67 billion and is projected to exceed USD 540 billion by 2035. Healthcare currently accounts for 30% of global data generation and is expected to grow at a compound annual growth rate (CAGR) of 36% by 2025, outpacing all other sectors. Hospitals alone generate an estimated 50 petabytes of data annually, with individual EHRs and medical imaging contributing roughly 80 megabytes of data per patient per year [4,5]. This magnitude of data highlights the urgent need for sophisticated ML and BDA systems to extract actionable insights effectively.

The emerging landscape of smart healthcare is shaped by a technological synergy that integrates blockchain, IoT, artificial intelligence (AI), and big data to enhance clinical outcomes, service personalization, and overall healthcare delivery. Wearable technologies have narrowed the gap in remote monitoring by providing continuous patient surveillance, enabling early detection, and timely intervention [6]. Predictive analytics are being employed to optimize resource allocation and forecast disease outbreaks, while AI-powered diagnostic tools improve the speed and accuracy of clinical decision-making. Telemedicine has emerged as a cost-effective and accessible mode of care, and blockchain offers a secure and interoperable framework for patient data exchange across institutions [1]. Furthermore, AI-driven automation is revolutionizing hospital operations, while personalized mobile applications that monitor vital signs and suggest lifestyle modifications are empowering patients to take a proactive role in their healthcare.

In the field of ML, algorithmic advancements have significantly enhanced the capacity to process complex healthcare data, detect patterns, and generate predictive models. Unstructured data types, such as medical imaging, genomic sequences, and clinical narratives, are being effectively managed through DL and natural language processing techniques, enabling improved real-time monitoring and operational efficiency [7]. These developments support the evolution of precision medicine and foster the transition towards a proactive and patient-centered healthcare ecosystem.

### Previous Reviews and Research Landscape

Some researchers and authors have made significant contributions in healthcare to manage voluminous data, taking advantage of ML and BDA. In [8], the authors explored how the COVID-19 pandemic highlighted the limitations of traditional healthcare and emphasized the need for smart, connected wearables powered by IoT. These devices generate vast health data, posing challenges in management and decision-making. BDA and ML have gained traction for improving IoTM applications. ML techniques were reviewed for big data analysis in healthcare, highlighting strengths, weaknesses, and research challenges to guide practitioners and policymakers. Semantic perception refers to the capacity of AI and ML systems to interpret and understand the meaning embedded within complex inputs, such as textual data, images, or sensor-generated signals, along with the contextual and relational dynamics among these data components [9]. In the context of healthcare, semantic perception enables systems to extract clinically relevant information from unstructured sources, such as physician notes or radiological images. This capability significantly enhances diagnostic accuracy and supports more informed clinical decision-making. In [10], the authors survey multimodal data-driven approaches in smart healthcare, covering decision-making processes, data fusion, and AI-driven transformations. This work presented semantic perception, entity association, and intelligent decision support, highlighting disease analysis, diagnosis, and privacy protection applications while addressing trends, challenges, and benefits in modern healthcare systems.

In [11], the authors presented ML and DL approaches in healthcare prediction, emphasizing their role in disease diagnosis from clinical data and images. It highlights AI-driven predictive analytics’ impact on accuracy, patient outcomes, and industry transformation while addressing challenges and the need for reliable, efficient methods in healthcare predictive analysis. In [12], the authors discussed how advancements in communication technologies and the Internet of Medical Things (IoMT) have revolutionized AI-driven smart healthcare. Traditional AI relies on centralized data processing, posing scalability and privacy challenges. Federated learning (FL) enables decentralized AI training across multiple clients without sharing raw data. This survey explores FL’s role in smart healthcare, covering recent developments, key applications, security considerations, and challenges. It also reviews FL-based healthcare projects and future research directions. In [13], the authors emphasized that detecting and classifying drug–drug interactions (DDIs) is crucial for preventing hospital-acquired conditions and improving smart healthcare. Given the vast medical literature, automated extraction using NLP techniques like information extraction (IE) and relationship extraction (RE) is essential. Traditional ML methods have limitations, while DL shows promise. They proposed SEV-DDI, a deep neural network model, enhancing precision in DDI classification and sentiment analysis to assess interaction severity for better clinical decision-making. In [14], the authors discussed how ML and DL have revolutionized healthcare, aiding in disease prediction, drug discovery, and medical imaging. They presented a comprehensive survey on ML and DL in smart healthcare, covering key advancements, applications, and future directions. We analyzed top journals and conferences, explored ML-DL integration, and highlighted research challenges and recommendations, providing a concise yet thorough overview of their impact on the healthcare industry. In [15], the authors discussed the role of BDA and FL in transforming healthcare by improving patient care and efficiency while addressing privacy concerns. FL enables collaborative data analysis without sharing sensitive information. This survey explored FL’s role in healthcare, its challenges, and future prospects, including real-time monitoring and predictive modeling. It offers insights into integrating FL with BDA, ensuring security, data quality, and interoperability for researchers, policymakers, and healthcare professionals. In [16], the authors explore the role of mobile health (m-Health) and big data in transforming healthcare. With vast structured and unstructured data generated daily, this paper examined big data issues in m-Health, focusing on communication, sensors, and computing. It explores integrating BDA with m-Health and the role of AI. Balancing innovation with clinical complexities requires stakeholder collaboration, shaping the future of intelligent, connected healthcare systems.

The swift convergence of big data and ML with smart healthcare systems represents a paradigm shift in medical data processing, interpretation, and application to clinical decision-making. As healthcare settings become increasingly digitized and networked, the capability to extract actionable insights from voluminous, disparate data sources becomes paramount. This review is a timely and extensive synthesis of how ML methods revolutionize healthcare delivery, improve diagnostics, facilitate real-time monitoring, enhance treatment personalization, and maximize operational efficiency.

Additionally, although there is a plethora of separate literature on AI and big data, few research studies explore their convergence in applied healthcare settings comprehensively, especially from the perspectives of real-world applications, cutting-edge technologies, and security concerns. By bridging such a gap, the current review synthesizes a cohesive framework that reflects recent developments and addresses limitations, challenges, and avenues for future research. It is hoped to advise researchers, developers, healthcare professionals, and policymakers on leveraging intelligent technologies to construct more scalable, secure, and patient-focused healthcare systems.

## 2. Methodology

This study adopts a narrative literature review methodology to address the research problem situated within the domains of ML, BDA, and IoT-enabled healthcare systems. Unlike systematic reviews, which follow a predefined protocol with structured data extraction procedures, narrative reviews provide a flexible framework for synthesizing knowledge across diverse sources and interdisciplinary perspectives.

To ensure transparency in the review process, the study outlines the general inclusion criteria that guided the selection of literature. Sources primarily comprised peer-reviewed journal articles indexed in reputable databases, including Scopus, Web of Science, IEEE Xplore, PubMed, and Google Scholar. Priority was given to recent publications (2018–2025), written in English, and directly relevant to smart healthcare applications, particularly those involving the integration of ML and BDA. The selection process was based on a comprehensive literature search prioritizing peer-reviewed journal articles, high-impact conference proceedings, and other scholarly publications published within the last decade, thereby ensuring coverage of the most recent advancements in the field. Studies were included if they addressed smart healthcare systems, ML techniques, and diagnostic applications while also engaging with ethical implications. Particular emphasis was placed on works offering unique insights, detailed case studies, or discussions concerning implementation challenges. This methodological strategy enabled the integration of both established knowledge and emerging perspectives across interdisciplinary domains.

It should be noted that no formal review protocol was registered, nor was a PRISMA flowchart employed, as this work does not constitute a systematic or replicable search. Instead, a broad thematic synthesis is presented, supported by illustrative examples from the literature.

## 3. Smart Healthcare Systems

**Figure 1:**
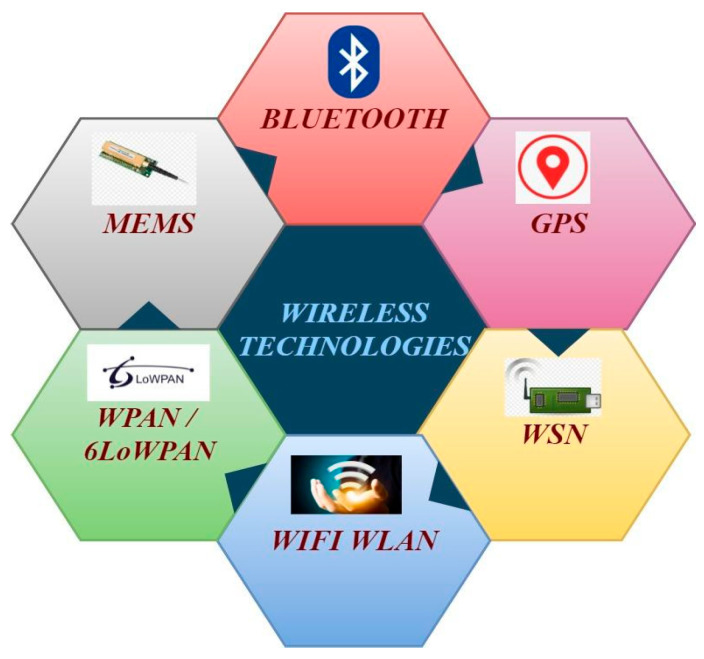
Wireless technologies: a significant contributor to smart healthcare systems. (Source: created by the authors).

Hospitals and other healthcare institutions today have much larger healthcare surveillance systems, and many nations worldwide are now seriously concerned about portable healthcare monitoring systems using emerging technology. In addition to tracking basic vital signs like body temperature and pulse rate, the device provided smart healthcare by measuring hospital room variables, including CO, CO_2_, and humidity levels. The application of information and communication technologies in the healthcare sector is referred to as “digital healthcare”. From merely keeping electronic patient records and offering patient online portals, electronic health care has advanced to allow for greater comfort and convenience in managing healthcare [17]. These days, it is known as connected health. The use of intelligent systems that support and enhance biological or medical processes and healthcare is accelerating due to recent advancements in IoT technology. In order to enable efficient patient monitoring even when patients are at home, smart health has developed from connected health, which uses wearable medical devices (such as heart rate monitors, glucose meters, smart watches, smart contact lenses, etc.) and IoT devices (such as implantable or ingestible sensors) in addition to traditional mobile devices (such as smartphones) [18]. By monitoring non-critical patients at home instead of in a hospital, smart healthcare could reduce the demand on medical resources like beds and physicians. It could be employed to facilitate access to healthcare for individuals residing in remote areas or enable older people to stay independently at home for longer. The core of the smart healthcare network is wireless technology, as illustrated in Figure 1. There are various wireless technologies like Wi-Fi, Bluetooth, 6LoWPAN, radio frequency identification (RFID), etc., that play a crucial role in the exchange of information between the numerous physical elements that make up the healthcare network [19].

In addition to giving people more control over their health at all times, it may encourage access to healthcare services while reducing the strain on medical systems. It is anticipated that smart health would lower healthcare costs while offering timely care for a range of illnesses. Enhancing the productivity of medical infrastructures and biomedical systems is among contemporary society’s most difficult undertakings. These include automated biomedical applications and monitoring of individuals in hospitals, appropriate drug-patient connections, and real-time monitoring of patients’ physiological data for the early detection of clinical deterioration [20].

The most promising technologies for smart healthcare systems are UHF, RFID, WSN, and smart mobile. Battery-powered WMSs are becoming more common as low-power sensor, processing, and communication technologies progress. According to Business Insider, more than 56 million wearable sensors were shipped worldwide in 2015, with that figure expected to rise to 123 million by 2018. WSNs are self-organizing ad hoc networks of small, minimal devices (motes) that interact multi-hop to provide monitoring and control capabilities in critical applications such as industrial, defense, residential, automotive, and medical. Most WSN motes are battery-powered computing platforms with analogue and digital sensors. These devices capture, preserve, and transmit physiological data in a quiet, productive, and efficient manner. Wearable sensors and body-area networks collect physiological signals such as ECG, SpO_2_, and blood pressure. Projects like CodeBlue and MobiHealth demonstrate secure data transmission for mobile health monitoring. Integrating sensor and EMR data using ML for structured analysis remains challenging but essential for intelligent, autonomous healthcare systems [21,22,23].

The growing adoption of a patented, decentralized, scalable, and immutable ledger system, underpinned by blockchain technology, for the secure and verifiable exchange of healthcare data. Blockchain facilitates the creation of tamper-resistant, time-stamped, and access-controlled medical records, which are retrievable solely by authorized stakeholders within smart healthcare systems. This architecture not only reinforces patient privacy but also significantly enhances data interoperability among healthcare institutions.

Furthermore, blockchain-enabled smart contracts offer the potential to automate insurance claims processing, streamline pharmaceutical supply chain management, and enforce patient consent protocols for data sharing. Collectively, these capabilities contribute to the establishment of a secure, transparent, and patient-centric digital health ecosystem. Table 1 summarizes recent advances in AI, IoT, and cybersecurity, showcasing smart healthcare innovations and future integration opportunities.

### 3.1. Smart Healthcare Framework

**Figure 2:**
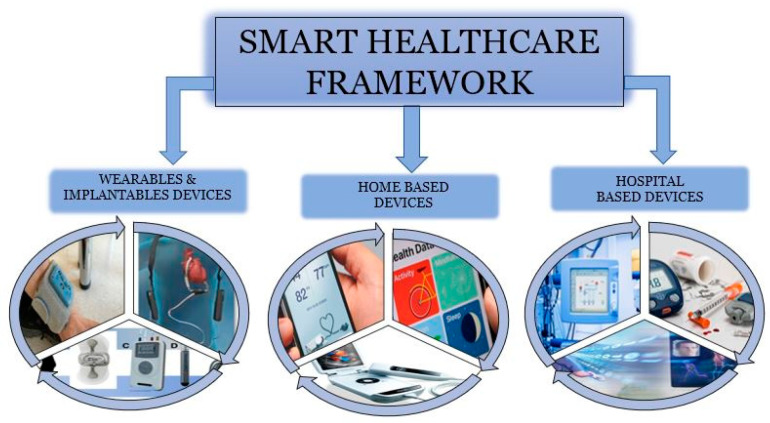
IoT-based smart healthcare equipment. (Source: created by the authors).

**Figure 3:**
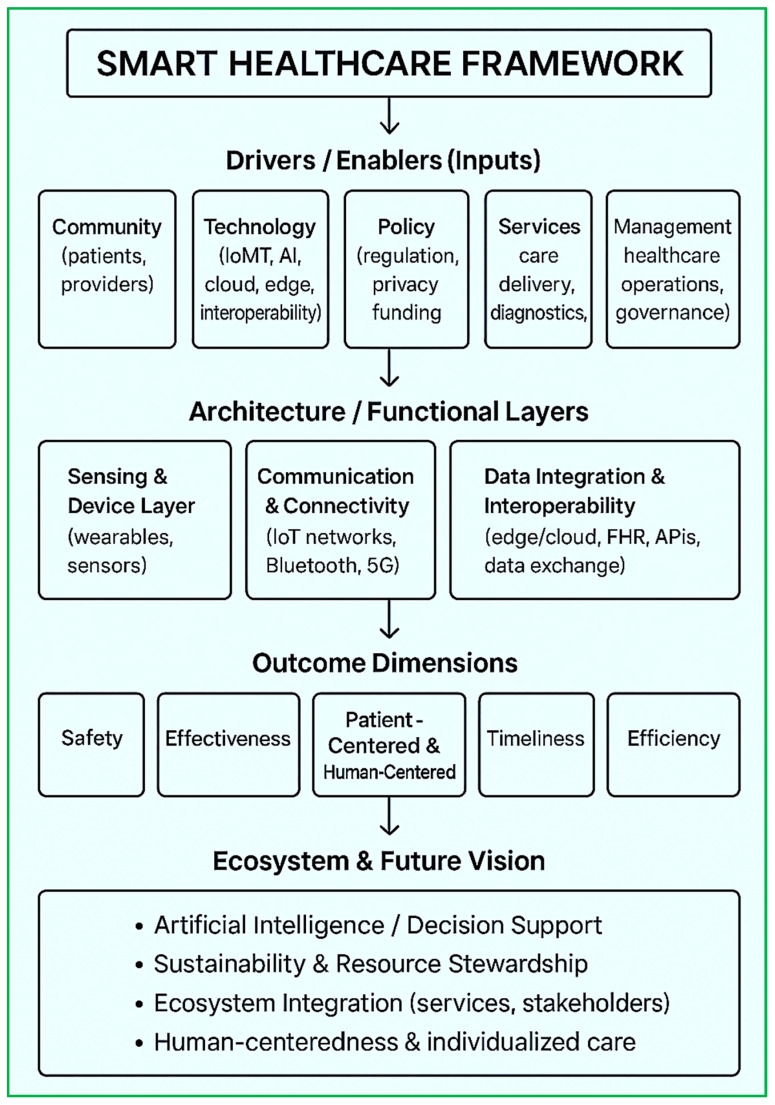
Smart healthcare framework dimensions. (Source: created by the authors).

Innovation is key to improving the quality of life across all age groups. With an aging population and advancements in AI and ML, the IoT offers a powerful means to monitor and deliver personalized healthcare. IoT devices collect and transmit patient data to caregivers and physicians, enabling timely and effective medical interventions [32,33,34]. With IoT-based healthcare equipment, patients are no longer confined to a single location or dependent on bulky medical apparatus. This innovation allows patients to move freely while being continuously and non-intrusively monitored. This also results in reduced cost monitoring. The following are the categorizations of IoT-based healthcare equipment/devices (shown in Figure 2) along with various aspects of the smart healthcare systems (Figure 3).

#### 3.1.1. Wearables IoT Devices

Wearable equipment is any small, wearable gadget that displays information to consumers and allows them to interact with it, whether that be via voice commands or manual input. It is frequently sold as a “casual clothing accessory”. Wearable technologies have steadily evolved as additions (e.g., bands, rings), smart clothes, body implants, and body penetrations (e.g., insulin pumps, artificial hearts). The development of multiple quasi-wireless sensors for measuring human physical parameters has expanded the potential for smart sensor network-based medical systems, wherein the sensors are wearable or portable. Innovative tools, such as surgically implanted and Wearable Healthcare Products, have permitted the interpretation and processing of physiologic information from anybody, anywhere, at any time [35]. Wearable technological advances include wireless transceiver modalities such as Bluetooth, Zigbee, infrared, broadcasting identification, Wireless internet, and relatively close communication. Such a platform enables wearables to link to other electronic objects (smartphones) for remote diagnosis and follow-up, resulting in improved quality of treatment. Surgically implanted and Wearable Biomedical Gadgets, which enable quasi-avoidance, accurate intervention, and ongoing medication of physical illnesses, are projected as critical pillars of modern healthcare. Wearable electronics have characteristics that concentrate on lower power consumption, compact size, manageability, and comfort of wear and use. Advances in wearable technology and significant analytics techniques make it possible to capture and modify medical application information in a genuine moment, which can strengthen quality healthcare by lowering injury rates, enhancing doctor–patient conversation, and discovering previously unseen monitoring and sensory features [36]. For example:

- A biker employing a vital sign system, specifically a wristband, is involved in an accident. The body sensor network detects the fall, which transmits an alarm to the city’s infrastructure. The technology replies by evaluating road congestion and dispatching ambulances through the most direct route. Furthermore, municipal traffic signals are automatically modified to shorten the time the ambulances take to reach the rider.
- Fitbit Inspire, a wearable fitness band, records all-day activity, relaxation, calorie counting, and breathing rate, among other things, and shows the results on an Android smartphone while storing the cloud to help the user achieve his or her chosen fitness goal. Google Fit, Fitbit Coach, Nike Training Club, Runtastic, and other mobile phone overall fitness applications track wellness and exercise regimens for individual requirements.

Table 2 summarizes recent advancements in smart healthcare, IoT security, and emerging technologies. It presents cutting-edge advancements in smart healthcare, IoT security, wearable technology, and military IoT applications. The research demonstrates how AI, ML, blockchain, edge computing, and next-gen networks are being integrated to improve security, efficiency, and real-time processing. Despite these advancements, challenges such as scalability, privacy risks, computational efficiency, and security vulnerabilities remain. Future research will likely focus on developing more resilient security frameworks, improving AI-driven analytics, and optimizing resource allocation in IoT-based systems. These findings emphasize the growing intersection of AI, IoT, and healthcare, shaping a future where secure, efficient, and intelligent systems will drive better healthcare outcomes, safer military operations, and more immersive Metaverse experiences.

#### 3.1.2. Home-Based IoT Devices

In-home remote monitoring apps enabled by the IoT are among the primary smartphone health (digital health) apps that allow effective preventive digital health treatments. Mobile-based IoT is a collection of software applications for healthcare solutions housed on digital devices that are based on sensors, computation, connectivity, and data and analytics. Home-based M-health is a mixture of telecommunications, imagery, detection, and human-machine interaction techniques that are used to diagnose and provide follow-up care without interfering with their daily lives. Telemonitoring applications use sensors and a residential hub to connect the patient to doctors and domain experts via cloud-based data offerings. Telemedicine provides the patient with assurance that their ailments (e.g., pulse rate, blood pressure, Blood oxygen saturation levels, and daytime sleepiness) are being tracked, and alarms may be created to notify their healthcare providers in actual environments. A wireless mesh network-based Remote health monitoring application framework incorporates wearable tech to help control various features of mobile computation devices, such as smartphones, smart iPads, and fitness trackers, among others [44,45]. The Open Smartphone Health (mHealth) initiative aims to prevent and treat serious illnesses regularly by leveraging data from medical systems on personal devices, such as smartphones. There seems to be a rapid growth in the availability of mHealth smartphone apps aimed at wireless connectivity and detection of various diseases, assisting patients in better managing their medical problems, and facilitating a successful life. The utilization of mobile gadgets contributes to more effective work performance. S-Health enables the remote monitoring of patients, encourages interaction between experts, household members, and individuals with health issues, and clarifies primary healthcare in three key ways: it provides access to user-oriented and personalized services and insights. Participant voice or visual home patient assistance via smartphones; IoT disaster preparedness; ambient-assisted living technology can deliver everyday situations, patient vital signs, and geospatial data; virtualized computerized medical monitoring, characteristic registration and categorization approaches; sensing smart gloves equipment for handicapped and developmentally disabled persons [46]. Social networking sites such as Facebook, Instagram, YouTube, and Google, which include content applications, journals, and weblogs, were made possible by Web 2.0. It presents a weight reduction app accessible via an Online social network. People discuss their objectives and achievements on the website, which provides anonymized and individualized assistance. Surveys are used to improve the platform’s offerings. By 2012, there would have been over 13,000 safety software applications on Apple’s iPhone. These programmers allow sufferers to capture and follow their critical physiological responses digitally regularly, resulting in satisfying customer consequences. Unless sufferers mention it, it is challenging for doctors to determine if their patients are taking their medications appropriately during a typical visit. This might have a negative impact on the patient’s recuperation. As a result, technologies for monitoring patients’ medication at home have evolved. iHomeHealth-IoT was a smart medicine box and smart pharmaceutical packaging. The smart box notifies the patient when it is time to take their medication [47]. The box also sends a text message to the doctor. For the aged patients, home is at the centre of the concept. It empowers patients via illness prevention, treatment, and consciousness of chronic medical conditions [48]. For example:

- Glycaemic monitoring apps supervise the patient’s glucose level. They can undertake a variety of actions relying just on the app, such as measuring the information in the device and/or the infrastructure as a service, displaying on connected phones, notifying caregivers, advising the general practitioner, and tracking to the health insurer, among other things.
- Electrocardiogram and cardiovascular monitoring applications use ECG, heart rate readings, and basic pattern recognition to follow the heart’s electrical activity. Depending on the app’s circumstances, they can forecast some fundamental problems, such as arrhythmias and cardiac ischemia, and notify people, clinicians, and caretakers.

Smart supervision, quick access to actual figures, and the transmission of on-demand judgments have laid the groundwork for a reliable integrated IoTM system that can guarantee more correct diagnoses, credible proof of treatment, fewer hospitalizations, and the best possible resource utilization regarding time, energy, and cost savings.

#### 3.1.3. Hospital-Based IoT Devices

Signaling is a technique employed in practically every element of surgery center care, from the most basic digital thermometers to complicated ultrasonically operating rooms. A sensor is a component that turns a physical measurement into a signal that an observer or equipment can analyze. There seems to be an interest in developing sophisticated medical equipment (e.g., heart rate devices, fructose meters, temperature monitoring, muscle mass scales, etc.). The LifeShirt Equipment is an example of a multi-sensor monitoring method for gathering, evaluating, and presenting a surgeon’s health data. It collects continuing patient data concerning data screenshots, which are typically acquired during the patient’s frequent appointments to the clinic or hospital [49,50].

The Smart-Health framework is intended to aid in the process of customizing and striving to improve the patient care prototype in a variety of ways, including the following:

- Concentrating on sensor technologies for health checks and appraisal techniques in the home and neighborhood atmospheres to minimize direct stress on healthcare environments and convert it to a digital dissemination of knowledge.
- Transforming the pharmaceutical procedure from a reaction to a proactive risk management strategy can dramatically reduce hospitalization costs for acute occurrences.
- Enhancing the personalized recommendations of the health and care system so that private citizens can supervise and recognize their potential risk characterization, preventive medicine intervention, and diagnosis, allowing patients to stay independent while being cared for, which has a strong positive influence on their psychological makeup and, as a result, their health status.
- Facilitating improved clinical maintenance and permitting the health service to prioritize sick people in the greatest need properly.
- Assisting self-care clinical techniques to observe health status and other varied metrics, in which these data are exchanged with a physician to conduct a diagnosis, either in person or by teleconsultation. Likewise, for mild conditions such as influenza, diagnosis can sometimes be computerized.
- Maximizing moment-in-time testing by lowering diagnostic time by eliminating the need to transfer specimens elsewhere to be analyzed. Computerized monitoring using blood pressure cuffs and electronic thermometers, for example, can assist the doctor in reviewing a medical history as statistics have been taken.

Table 3 summarizes a comparison of all three types, i.e., wearable, home-based, and hospital-stationed smart devices, from ML and big data aspects.

## 4. Machine Learning and Big Data Applications in Smart Healthcare Diagnostics

### 4.1. Overview of Machine Learning

ML, a practical subset of AI, enables systems to learn from data and make predictions or decisions without the need for explicit programming. These algorithms identify patterns within historical datasets and generalize this learned knowledge to unseen data, rendering them particularly suitable for healthcare contexts, which frequently involve complex, high-dimensional information [51].

**Figure 4:**
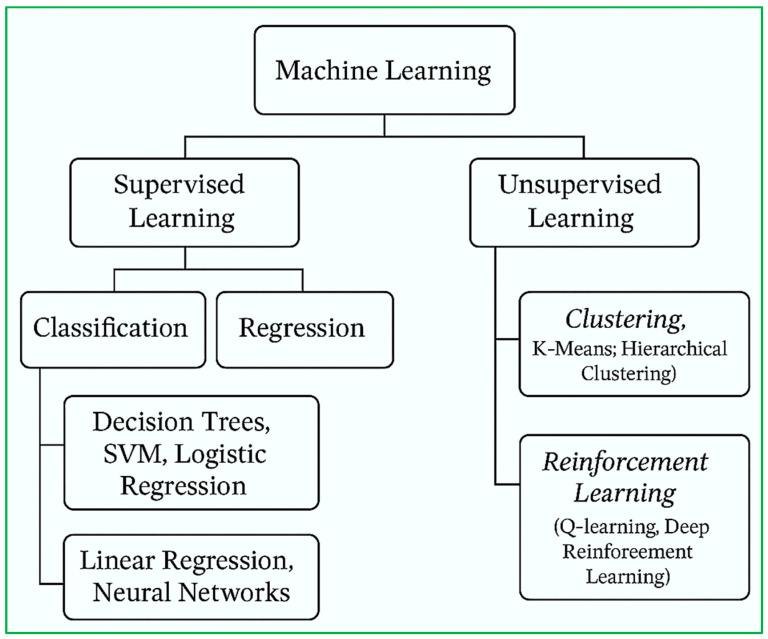
Taxonomy of machine learning methods. (Source: created by the authors).

ML methodologies can be broadly classified into three primary categories: supervised learning, unsupervised learning, and reinforcement learning, as illustrated in Figure 4.

Supervised learning involves training models on labeled datasets. This category encompasses two major subtypes [52]:

Classification, which aims to predict categorical labels (e.g., presence or absence of disease) [53], commonly employs algorithms such as decision trees, support vector machines (SVMs), and logistic regression.

Regression, which is used for predicting continuous outcomes (e.g., blood glucose levels), utilizes techniques like linear regression and neural networks.

Unsupervised learning deals with unlabeled data, aiming to uncover hidden structures or intrinsic patterns. Clustering methods such as K-means and hierarchical clustering group similar data points based on inherent similarities without prior knowledge of class labels [54].

**Figure 5:**
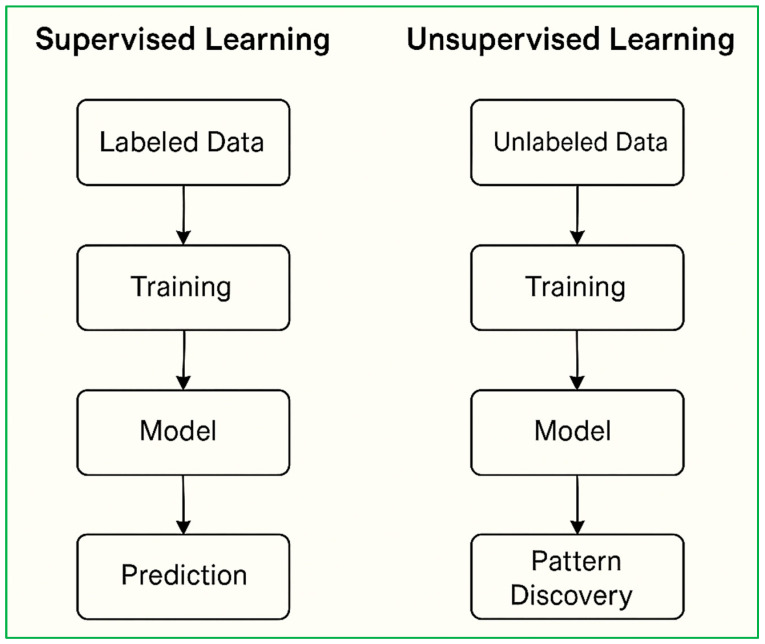
Workflow of supervised vs. unsupervised machine learning. (Source: created by the authors).

Reinforcement learning refers to the process of learning optimal actions within a dynamic environment to maximize cumulative rewards or minimize penalties. Algorithms such as Q-learning and deep reinforcement learning are increasingly being investigated for their potential in adaptive, real-time healthcare decision-making [55]. Figure 5 depicts the taxonomy of machine learning methods categorized by learning approach, with representative algorithms for each.

Supervised and unsupervised learning also diverge significantly in terms of workflow, as outlined in Figure 5. In supervised learning, the pipeline follows a clear progression: labeled data, model training, and prediction generation [54].

In unsupervised learning, the flow is: unlabeled data, model training, and pattern discovery.

The distinction in workflow between these two paradigms, as visualized in Figure 5, highlights their respective data requirements and end-goals. This foundational understanding of ML methodologies serves as a conceptual precursor for the application of these approaches in smart healthcare systems [56].

### 4.2. Big Data in Smart Healthcare

The advent of big data in healthcare, powered by technologies such as IoT, cloud computing, and data mining, has enabled real-time patient monitoring, predictive diagnostics, and personalized care delivery. These innovations are transforming traditional healthcare into a proactive, data-driven ecosystem. The main challenge facing healthcare systems is finding ways to detect, collect, analyze, and manage data to assist patients in understanding new diseases and medications, predict outcomes early, and make decisions in real time. The healthcare business generates a substantial amount of data, including patient records, drug information, experimental results, consultations, and real-time data that is both simultaneous and immediate. This data must be processed and handled. Therefore, big data encompasses a range of methods, tools, and strategies for data collection (structured or unstructured), information extraction, organization, analysis, management, and, most importantly, utilization for future purposes. Automating medical data changes the dimensionality of data while also increasing the size of data and the importance of data analytics. In a digitalized healthcare context, healthcare data refers to the vast data that is harder to manage with traditional database management systems, including health centers, fitness trackers, and social platforms. Big data assists in digitizing and integrating information to be user-friendly and easily retrieved. Nevertheless, big data may help us discover hidden patterns, correlations, and cross-correlations in the data it accumulates. BDA is a tool for analyzing a massive amount of digital data associated with patient healthcare and well-being that is exceedingly varied and challenging to evaluate using standard software or hardware [57].

**Figure 6:**
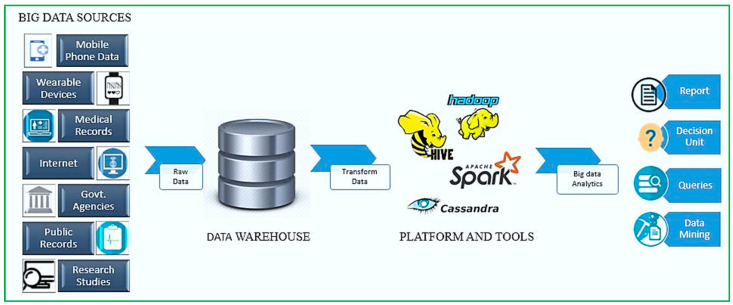
Data analytics framework for big health data. (Source: created by the authors).

Healthcare BDA has especially come to the forefront as a viable technique for addressing difficulties in a variety of healthcare domains (Figure 6). The primary goal of governments throughout the world is to enhance healthcare facilities while lessening medical expenditures. Big data offers doctors, biochemists, and patient care specialists a fantastic chance to make computational decisions that will eventually strengthen patient care. Big data in healthcare has the potential to transform the medical industry by allowing for early illness identification utilizing appropriate analytical tools and methodologies, as well as by combining and interpreting health-related data in a precise way.

The development and implementation of ML technologies facilitate the efficient utilization of Big Data in Healthcare. Techniques based on ML are sometimes used alternately with methodologies based on AI. The introduction of artificial intelligence and data analytics, coupled with the increasing interconnection opportunities of medical devices, i.e., the IoT, has created a significant opportunity for microbiologists to predict and allocate accurate and effective resources to the appropriate patients. Patient information includes recorded signals such as ECGs, pictures, and videos. Healthcare professionals have only just begun to transfer such healthcare data into EHRs. Attempts are being made to digitize patient records from pre-EHR period entries to support standardization by converting static photos into machine-readable text [58].

### 4.3. Convergence of Machine Learning and Big Data

**Figure 7:**
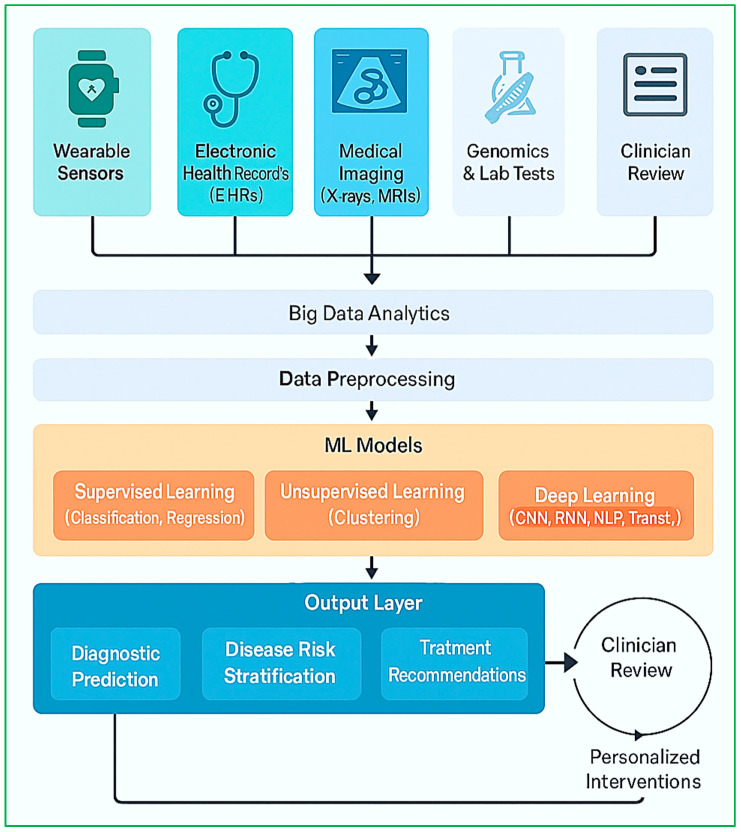
Machine learning diagnostic insight pipeline: from data acquisition to clinical decisions. (Source: created by the authors).

The convergence of ML and BDA in healthcare has resulted in tremendous improvements in diagnostic performance, disease forecasting, patient tracking, and decision-making. This convergence allows intelligent healthcare systems to transcend conventional diagnostic processes by tapping into enormous amounts of structured and unstructured data, such as EHRs, sensor readings, medical images, genomic data, and operational hospital data. The capability to analyze such data in virtual real time accelerates early detection, risk stratification, customized treatment planning, and operational effectiveness. As illustrated in Figure 7, the diagnostic insight pipeline combines multimodal health information, from wearables and EHRs to genomic data and medical images, into a machine learning pipeline that facilitates predictive diagnostics, risk stratification, and individualized decision-making. A feedback mechanism allows for iterative model refinement through clinical outcomes, increasing trust, accuracy, and interpretability.

Recent work has illustrated how various ML methods, covering ensemble classifiers and support vector machines to DL models and federated learning, can be efficiently used in multiple clinical fields [59,60]. These are cardiology, ophthalmology, endocrinology, nephrology, hepatology, maternal and geriatric care, intensive care, and hospital-wide operations. Big data sources such as real-world evidence, wearable sensors, IoT-enabled devices, and cross-institutional EHRs offer the size and complexity to enable these models to execute solid diagnostic tasks. Yet, with these opportunities come challenges in data privacy, interpretability of algorithms, generalizability of models, and clinical integration.

Table 4 presents some of the most important recent contributions in the area, highlighting the range of diagnostic applications enabled by ML and big data. The examples vary from predictive models for diabetes complications based on XGBoost to liver fibrosis detection through hybrid classifiers, and smart hospital transformation programs utilizing ERP-integrated AI systems. Notably, several studies point to privacy-preserving designs and non-invasive diagnostic substitutes, respectively, that are essential in scalable clinical rollout.

## 5. Machine Learning for Voluminous Healthcare Data: Real-World Case Studies

Smart healthcare systems increasingly rely on ML techniques, which broadly fall into three primary categories: supervised, unsupervised, and reinforcement learning. These techniques enable the analysis of large-scale structured and unstructured healthcare data, facilitating real-time decision-making, anomaly detection, and the delivery of personalized care.

Supervised learning models are predominantly used for tasks such as disease classification and outcome prediction [81,82]. Unsupervised methods are effective for clustering patients based on symptomatology or behavioral patterns, while reinforcement learning is particularly suited for optimizing treatment pathways in dynamic clinical environments.

The implementation of these methods is particularly crucial in domains such as remote patient monitoring, diagnostic decision support, and clinical workflow automation. ML offers an indispensable toolkit for advanced analytics, empowering healthcare stakeholders to extract insights from vast and heterogeneous datasets, insights that are unattainable through conventional statistical methods alone.

**Figure 8:**
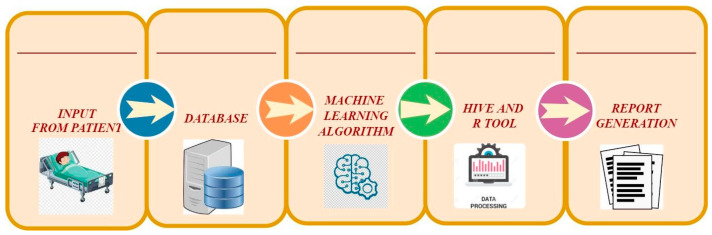
ML for data-intensive healthcare applications. (Source: created by the authors).

Applications of ML in healthcare span various data-intensive tasks, including predictive analytics, anomaly detection, and clinical decision support, as illustrated in Figure 8. A comprehensive overview of ML applications and their associated Big Data challenges, from both clinical and operational perspectives, is provided in Table 5.

### 5.1. Real World Case Studies

Diagnostics, treatment planning, and patient care have all significantly improved due to the fundamental transformation of medical data utilization brought about by integrating BDA and ML into healthcare systems. By utilizing big datasets and sophisticated algorithms, healthcare providers can find previously undiscovered patterns and insights, which makes it possible to provide more individualized and efficient treatment. This section presents a collection of real-life case studies demonstrating the revolutionary impacts of big data and ML across various healthcare settings, highlighting the practical applications and benefits of these two technologies.

#### 5.1.1. UK Biobank: Advancing Global Medical Research

The UK Biobank, a central biological database and research tool, includes five hundred thousand people’s anonymized genetic and health data. It was established to enhance the prevention, diagnosis, and treatment of various serious and life-threatening diseases. This resource is accessible worldwide to authorized researchers conducting essential studies on prevalent illnesses. The use of BDA and ML methods has allowed scientists to identify patterns and relationships that deepen our comprehension of complex diseases [83].

The UK Biobank prioritizes data security and privacy, ensuring that participant information remains safeguarded while enabling transformative research. By offering a robust data repository, it supports research initiatives that aim to improve health outcomes and foster personalized medicine developments. The Biobank is continually adapting, integrating new types of data, and broadening its scope to support diverse health research endeavors [84].

#### 5.1.2. NVIDIA and GE HealthCare: Transforming Diagnostic Imaging

NVIDIA and GE HealthCare have partnered to develop AI-powered X-ray and ultrasound devices to promote innovation in autonomous imaging. The partnership will enhance diagnostic imaging capabilities by applying NVIDIA’s AI technologies, enabling more accurate and effective diagnoses. Clinical operations will be made simpler and patient outcomes will be improved by integrating AI into medical imaging processes. This partnership serves as an example of how BDA and ML may be used to revolutionize healthcare delivery. Healthcare providers can provide faster and more accurate diagnoses because of AI, which will eventually improve patient care. The collaboration also emphasizes the value of cross-industry collaborations in spurring innovation and enhancing healthcare systems [85].

#### 5.1.3. Healthcare Platform Utilizing Big Data Analytics (BDA)

A medical platform that combines big data analysis and ML was able to detect diseases with more than 95% accuracy and lower costs by 90% in comparison with conventional hospital procedures. The platform’s architecture utilizes distributed computing and sophisticated algorithms for efficiently processing large volumes of healthcare information. Through this method, real-time analysis is facilitated and clinical decision support is offered, which improves patient outcomes. Such platforms can improve access to quality healthcare services by providing correct diagnostics at one-sixth the cost. The adaptability and scalability of the system make it easy to implement the system in many different healthcare settings, leading to improvements in patient care on a large scale [86].

#### 5.1.4. Big Data in Oncology Drug Development

Integrating BDA in oncology drug development has shown immense potential in advancing precision medicine. By analyzing large-scale biological experiments, clinical trials, and medical records, researchers can identify biomarkers and genetic profiles associated with specific cancer types [87].

This information enhances patient outcomes and treatment efficacy by facilitating the development of tailored medicines. Big data also expedites the drug development process by making it possible to identify prospective medication candidates and forecast their success probabilities. This strategy has decreased the time and expense of introducing novel cancer medicines to the market. Furthermore, the insights gained from big data analyses support the personalization of cancer care, ensuring that patients receive therapies tailored to their unique genetic makeup [88].

#### 5.1.5. Oncora Medical: Streamlining Oncology Workflows

Oncora Medical integrates big data, automation, and ML to streamline workflows for oncologists to provide faster and more individualized treatment to cancer patients. Its software aggregates information from multiple clinical systems, aligns it to standardized ontologies, and extracts key information automatically to generate a real-time, research-grade data store. This complete data management aids in data-driven clinical decisions and improves treatment planning [89].

Through AI-driven tools, Oncora Medical’s product line makes it easier to document and analyze oncology cases, enhancing the speed and precision of cancer treatment. The Cancer Registry Assistant, for instance, makes it easier for healthcare providers to streamline quality control for precise data gathering and reporting. Such innovations are a perfect example of the potential that big data and ML can have in revolutionizing oncology practice [90].

#### 5.1.6. IQVIA’s NLP Data Factory for Population Health

IQVIA’s NLP Data Factory is transforming the way healthcare organizations manage unstructured information [91]. By aggregating significant insights from clinical notes, lab results, and other unstructured sources, the platform helps healthcare payers enhance population risk stratification [92]. This innovation has made it possible to more effectively identify high-risk patients, enabling timely interventions and improved health outcomes.

NLP Data Factory analyzes millions of records in an hour, making the data analysis process far less time- and resource-intensive. By converting unstructured data into actionable intelligence, IQVIA’s solution enables smarter decision-making and better patient care [93].

#### 5.1.7. Digital Health Platform in Colombia

A Colombian healthcare company has developed a digital health platform integrating high-level analytics with operational, clinical, and business data sources. The goal of the integration is to improve population health management decision-making. By combining various data sources, the platform offers an overall overview of patient populations, allowing for more efficient health interventions.

Its utilization of big data and ML methods enables real-time analysis and predictive modeling. It allows proactive healthcare approaches, including early disease detection and resource allocation. The Colombian case study shows the potential for digital health innovations to augment healthcare delivery, especially in resource-poor contexts [94].

#### 5.1.8. BigQuery ML for Diabetes Prediction

Scientists have used Google’s BigQuery ML to create a diabetes prediction model, showing the platform’s potential to make healthcare analytics more accessible. By allowing ML models to be created through ordinary SQL queries, BigQuery ML reduces the threshold for healthcare experts who lack advanced programming experience. This opens up ML tools for broader use in clinical environments.

The diabetes risk prediction model performed excellently, demonstrating BigQuery ML’s effectiveness in processing big data. Integration into current healthcare workflows enables real-time risk calculation and tailored patient care. The case study emphasizes the value of accessible ML capabilities in promoting data-driven healthcare solutions [95].

#### 5.1.9. HealthEdge: Predicting Type 2 Diabetes

HealthEdge is an intelligent health platform that utilizes ML within an integrated cloud, edge, and IoT computing system to forecast Type 2 Diabetes. Through real-time examination of medical sensor and device data, the platform can evaluate unique risk factors. Early detection and intervention are significant in managing chronic conditions such as diabetes.

The architecture of the framework supports efficient data processing at the edge, minimizing latency and conserving bandwidth. ML models are trained in the cloud and pushed to edge devices, enabling scalable and real-time healthcare solutions. HealthEdge demonstrates how the convergence of cutting-edge technologies can improve disease prediction and patient outcomes [96].

#### 5.1.10. AI Predicting 10-Year Heart Disease Risk

Researchers have developed a DL model based on a single chest X-ray that can predict a patient’s 10-year chance of dying from a heart attack or stroke. Over 147,000 chest X-rays from more than 40,000 persons were used to train the algorithm, which identified patterns associated with cardiovascular risk. The noninvasive method offers a possible instrument for heart disease prevention and early diagnosis.

The predictions by the AI model were comparable to conventional risk assessment tools that involve blood work and blood pressure readings. This technique may simplify risk stratification and facilitate timely intervention using already available imaging information. The research brings out the value of AI in making diagnostic accuracy possible and improving care for patients in cardiology [97].

The diabetes ML predictive model leverages key patient characteristics, specifically age, body mass index, blood glucose levels, family medical history, and lifestyle patterns, to identify individuals at elevated risk of developing diabetes at an early stage. This predictive capability enables clinicians to implement timely, targeted interventions aimed at preventing or delaying the onset of diabetes-related complications. Such interventions may include personalized nutrition plans, tailored exercise regimens, and continuous glucose monitoring programs, all of which contribute to reducing the incidence and progression of diabetes.

Table 6 compares ten real-world case studies showcasing BDA and ML in smart healthcare systems. It summarizes essential elements, including the participating organizations, application domains, technologies employed, and the results obtained, demonstrating these technologies’ various applications and advantages in improving healthcare delivery.

The real-life case studies demonstrate the revolutionary effect of BDA and ML in different healthcare fields. These applications, from disease prediction and diagnostic imaging to population health management, show how data-driven technology improves decision-making, lowers expenses, and personalizes patient care. One takeaway is the scalability and flexibility of these technologies to both developed and resource-poor environments, affirming their global applicability. In addition, partnerships between health organizations and tech giants (e.g., NVIDIA, Google Cloud) are speeding up innovation and simplifying clinical workflows, setting the stage for future digital health ecosystems.

The case studies highlighted BDA and ML’s revolutionary potential in various aspects of healthcare. These technologies are transforming the healthcare landscape, from enhancing diagnostic imaging and optimizing oncology workflows to improving drug development and population health management. Healthcare professionals can deliver more personalized, efficient, and effective care by utilizing massive datasets and complex algorithms. As BDA and ML continue to integrate, they promise further innovations that will enhance patient outcomes and revolutionize healthcare delivery.

### 5.2. ML-Driven Innovations in Disease Diagnosis and Early Detection

ML has revolutionized disease diagnosis by rapidly and accurately detecting intricate health states from large and diverse datasets. These developments cover various diagnostic modalities, including medical images, genomic sequences, clinical text, wearable sensor signals, and electronic health records. Unlike conventional rule-based diagnostic systems, ML models, particularly DL architectures, can automatically learn patterns and features from data without explicit programming [98]. This makes them well-suited to handle non-linear, high-dimensional healthcare data.

**Figure 9:**
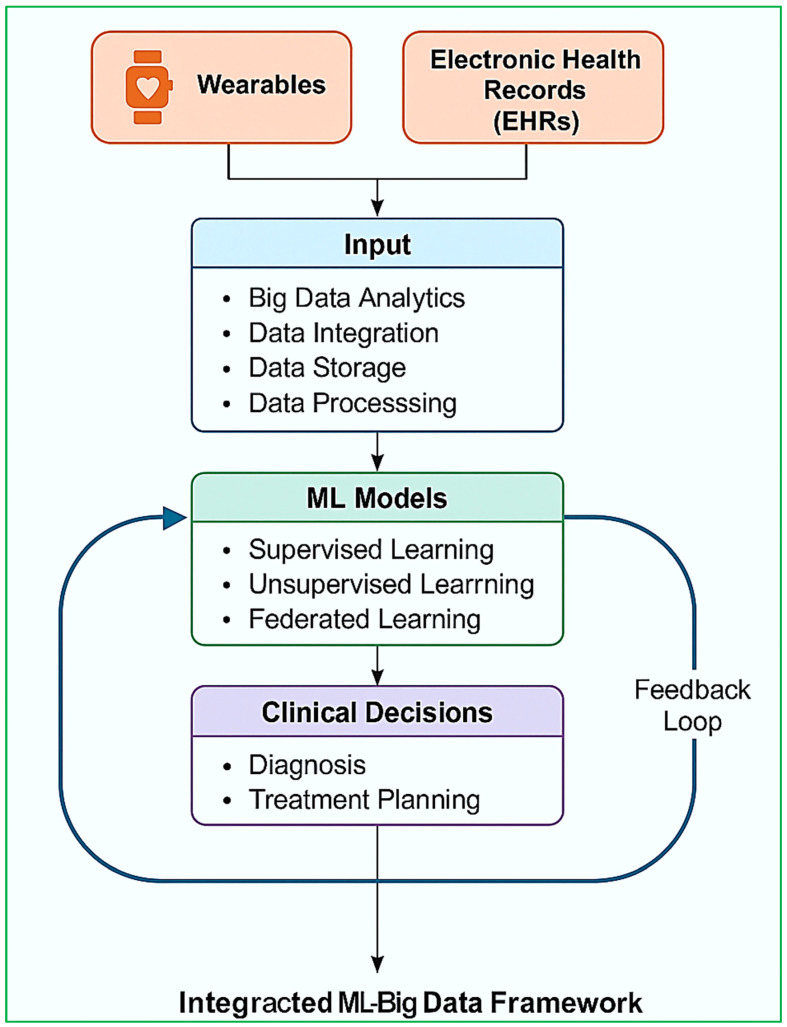
Integrated ML–big data framework for smart diagnostics: from acquisition to decision support. (Source: created by the authors).

Figure 9 depicts the integrated ML–big data framework for smart diagnostics. It reflects the end-to-end process from data acquisition through wearable sensors and EHRs, big data analytics, and machine learning models to final clinical decisions. The framework has a feedback loop mechanism, allowing continuous model updating and personalized treatment planning, thus improving the accuracy and responsiveness of healthcare systems.

#### 5.2.1. Medical Imaging and Radiology

CNNs have transformed radiological diagnosis by achieving high accuracy in detecting conditions [99]. For example, a CNN model achieved 95.19% accuracy in detecting pneumonia from chest X-rays, demonstrating its suitability for real-world clinical use [100]. In another example, a deep CNN ensemble produced an accuracy of 93.91% in diagnosing pneumonia, demonstrating the effectiveness of state-of-the-art ML methods in medical imaging [101].

#### 5.2.2. Natural Language Processing in Clinical Text

Natural language processing (NLP) has been a central innovation in extracting unstructured clinical notes and pathology reports to detect diseases at an early stage. Models based on the Transformer, such as ClinicalBERT and BioBERT, have gained traction in handling clinical documentation, facilitating diagnostic cues extraction, and supporting [76]. NLP develops automated coding and documentation procedures. Such models enable early detection of conditions like Alzheimer’s by analyzing electronic health records and clinical notes [102].

#### 5.2.3. Large Language Models (LLMs) in Healthcare

LLMs represent an advanced class of DL architectures trained on extensive text corpora, enabling them to understand and generate human-like language. Prominent examples include BERT, GPT, and domain-specific variants such as ClinicalBERT, all of which have demonstrated exceptional performance in tasks such as text classification, question answering, and summarization [103].

In the healthcare domain, LLMs hold particular promise in extracting meaningful clinical insights from unstructured textual data sources, including physician notes, discharge summaries, and pathology reports. By leveraging their capacity to identify linguistic patterns and contextual relationships, LLMs enhance clinical decision support systems through anomaly detection, pattern recognition, and synthesis of relevant patient information. This, in turn, contributes to faster and more accurate diagnostics, informed treatment planning, and improved risk stratification [104].

#### 5.2.4. Wearable Devices and Continuous Monitoring

Wearable technology is now essential to continuous health monitoring, giving instant physiological data that can be processed using ML to enable early disease detection. For instance, smartwatches have been used to identify early symptoms of Parkinson’s disease by tracking fine movement changes in movement patterns, potentially allowing for earlier interventions. Furthermore, AI-enabled wearable sensors have been created to track health markers in real-time and provide actionable data-driven insights that enable early disease detection and personalized interventions [105].

#### 5.2.5. Neurodegenerative Diseases and Early Detection

ML methods have been up-and-coming in the early diagnosis of neurodegenerative disorders like Alzheimer’s [106]. Experiments have proved that ML models can forecast the conversion from mild cognitive impairment to Alzheimer’s disease dementia by evaluating neuroimaging data and other biomarkers [107]. These predictive functions allow for early interventions and improved management of disease progression.

#### 5.2.6. Integration and Future Prospects

The incorporation of ML across different diagnostic fields is transforming the early detection of disease. It allows for proactive and tailored interventions, minimizes diagnostic delay, and improves outcomes in various areas of medicine. Ongoing research is needed to enhance model interpretability, resolve data bias, and guarantee safe deployment in real-world clinical settings [108].

## 6. Major Challenges Faced in Processing Huge Healthcare Data

### 6.1. Data Challenges

Big data challenges are frequently conceptualized through the lens of the “five Vs”: Volume, Velocity, Variety, Veracity, and Volatility, each representing a critical dimension of complexity in managing large-scale data environments.

- Volume: The sheer quantity of data generated by organizations, often reaching hundreds of terabytes or even petabytes, stems from routine business operations and regulatory obligations. In the healthcare domain, this data proliferation quickly leads to saturation, wherein the proportion of actionable or relevant information diminishes. This phenomenon gives rise to what is termed the “blind zone”: a segment of data characterized by unknown or unexamined facts that may be either inconsequential or critically informative.
- Variety: The exponential growth of sensor technologies, IoT devices, and diverse communication platforms has resulted in a highly heterogeneous data landscape. Data today spans structured formats (e.g., relational databases), semi-structured formats (e.g., XML or JSON with identifiable markers but lacking a rigid schema), and unstructured formats (e.g., textual data, multimedia, and user-generated content from websites and social media). This diversity presents formidable challenges for integration, storage, and analysis across systems.
- Velocity: The velocity dimension refers to the rapid rate at which data is generated, transmitted, and analyzed. Modern data streams require real-time or near-real-time processing due to their ephemeral value. In contexts such as healthcare, where clinical decisions are time-sensitive, the inability to process high-speed data flows in transit can result in missed opportunities for timely interventions.
- Veracity: Veracity pertains to the reliability, accuracy, and quality of data. Issues such as misinformation, incomplete records, and noise complicate the extraction of meaningful insights. Given the scale of big data, ensuring data veracity becomes a significant hurdle, particularly in fields like healthcare, where analytical precision is paramount. Compounding this issue is the shortage of highly skilled data scientists, professionals adept in data mining, transformation, interpretation, and innovation, whose expertise is often prohibitively expensive and challenging to retain.
- Volatility: Volatility describes the degree to which data can be deemed reliable over time and the duration for which it remains relevant or valid within a system. In an era increasingly reliant on data-driven insights, understanding the temporal sensitivity of data, when it becomes obsolete, is essential for maintaining the integrity and usefulness of information used in decision-making processes.

Collectively, these five dimensions emphasize the multifaceted challenges inherent in the effective utilization of big data, particularly in domains where precision, trustworthiness, and timeliness are critical [109,110].

### 6.2. Process Challenges

Data provenance refers to the documentation of the origin and transformation of data, enabling the tracking of its evolution and informing decisions regarding its suitability for future processing. In the context of big data, datasets must be horizontally prepared through standardized formatting and consistent extraction processes to facilitate analysis. However, maintaining such a data pipeline can be labor-intensive and complex. Therefore, effective data integration, encompassing the consolidation of disparate systems, operations, and databases, is critical for ensuring analytical productivity. To support this, mechanisms for aggregation, automation, and system compatibility must be in place, thereby contributing to the development of a robust and reliable analytical framework Similar challenges regarding algorithmic deployment, optimization strategies, and integration across complex data environments have been observed in adjacent domains like manufacturing, where ML and DL have faced barriers related to model generalization and real-time inference [111].

### 6.3. Management Challenges

Driven by the rapid proliferation of data vulnerabilities, additional big data challenges have emerged, notably in the areas of connectivity, cost, data privacy, and regulatory compliance. These concerns are particularly pronounced in domains such as healthcare, where the protection of personal information is paramount. The improper use or integration of personal data, especially when aggregated from multiple disparate sources, raises serious ethical and legal concerns.

For example, the adoption of cloud technologies to facilitate the sharing of healthcare data introduces potential risks. While cloud-based platforms can enable the pooling of patient data across multiple providers to enhance diagnostic capabilities and preventive care, they also introduce significant vulnerabilities. In contexts where pharmaceutical data security is limited, the reliance on third-party cloud infrastructure compounds the risk of data breaches and unauthorized access.

The increasing reliance on big data analytics has led many enterprises to construct expansive computing environments capable of storing, integrating, and analyzing petabytes of information. These centralized data repositories present attractive targets for malicious actors, including organized cybercriminal groups, due to the potential for high-value data exfiltration, often described as the “crown jewels” of an organization.

A further operational challenge lies in selecting appropriate technological frameworks. Enterprises must evaluate whether open-source platforms such as Hadoop are sufficient for their needs, or whether commercial alternatives, including Cassandra, Cloudera, Hortonworks, and MapR, offer better scalability, security, and support for enterprise-grade big data management. Balancing performance, cost-effectiveness, and compliance with privacy regulations remains a complex but critical endeavor in the modern big data landscape [112].

## 7. Existing ML-Based Big Data Solutions in Managing Healthcare Data

**Figure 10:**
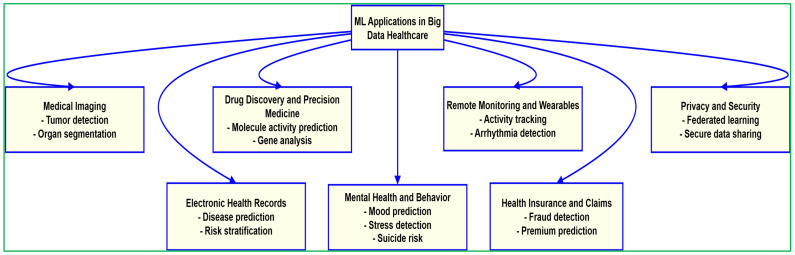
ML applications in voluminous healthcare domains.

The advent of ML has dramatically enhanced healthcare activities and services, giving rise to the new discipline of “smart healthcare”. ML-based algorithms are frequently more precise than trained practitioners, particularly in pathology and imaging. The most common use of ML in the healthcare field is sickness diagnosis, such as remote healthcare, diagnosis and treatment, healthcare monitoring, treatment adherence, and body sensors. Intelligent software is predicted to aid radiologists and clinicians in assessing patients in the coming years, and ML will transform medical knowledge and education [113]. Medical healthcare has developed as an intriguing application field for ML models, with these models already achieving human-level performance in clinical pathology, radiography, ophthalmology, and dermatology. The primary applications of healthcare that can benefit from ML approaches are as follows (Figure 10).

### 7.1. Diagnosis and Treatment

In smart healthcare, various illness detection technologies are connected to computerized decision-making. The categorization system may someday take the role of medical professionals in identifying illnesses if it performs well in terms of overall reliability and testing time. The most notable and outstanding product in clinical decision support systems is IBM’s Watson10, an artificial cognitive system that provides the best answer by thoroughly analyzing all medical and literature data. Watson can answer health-related questions in everyday and medical applications in the healthcare industry. Scientific papers, patents, medication and disease terminologies, drug testing, electronic health records, laboratory and radiology data, genomic data, claims data, and online social data are just a few medical resources it can scan and assess. Physicians may use clinical decision support systems to offer expert guidance based on algorithms to improve diagnostic performance, reduce the number of erroneous diagnoses and misinterpretations, and allow patients to get prompt and efficient treatment.

Among the most complex non-communicable diseases to treat at this time are dementia and Alzheimer’s disease. Alzheimer’s disease diagnosis, dementia diagnosis, Alzheimer’s disease-related area identification, mild cognitive impairment patient conversion to Alzheimer’s disease prediction, and Alzheimer’s disease-related gene discovery are among the uses. Fourteen distinct machine-learning algorithms were used. The patient’s health and illness status are more correctly defined using smart diagnosis, which aids in developing a customized treatment plan, and specialists have endorsed the software [114,115].

### 7.2. Medical Imaging

These days, medical imaging is automated. Several limitations need to be resolved before they can be utilized effectively in healthcare. When combined with radiological imaging, machine learning approaches help identify these sickness symptoms in people. “Medical imaging” refers to various techniques to provide visual depictions of the human body’s interior for research, diagnosis, and treatment. This eliminates the need for the conventional clinical standard of surgical therapy. There is an increased risk of infections, strokes, and other complications when any portion of the human body is surgically accessed. ML in medical imaging includes positron emission tomography (PET) scans, ultrasound imaging, computerized axial tomography scans, and magnetic resonance imaging. It has been exposed that the established automated models may be utilized to identify various chest-related disorders, such as TB and pneumonia [116,117].

The most prevalent use of contemporary ML technologies in healthcare is computer-automated detection (CAD), especially in identifying lesions seen in mammograms, brain scans, and other body scans. Breast imaging has shown the most success with CAD. Computer-aided detection (CADe), in which the machine alerts the radiologists to prospective lesions, and computer-aided diagnosis (CADx), in which the computer estimates the chance that a lesion is cancerous, are both examples of CAD.

The outcome of these imaging methods is a collection or sequence of pictures that a radiologist must interpret and diagnose. ML approaches quickly improve their ability to anticipate and locate pictures that may signal a disease condition or primary concern.

### 7.3. Drug Discovery and Development

ML techniques offer tools to improve discovery and decision-making for well-defined topics with high-quality data. ML can lower medication development and research failure rates and enhance data-driven decision-making. The most widely used method in drug development is to design drugs (small molecules, enzymes, antibodies, or more recent modalities like short RNAs or cell therapies) that alter a biological target’s functioning to impact the disease state. ML holds a lot of promise in the study of fundamental biological concepts to find therapeutic opportunities through alternative modalities or new targets [2]. The investigation of genetic variability in splicing signals is one facet. Drug development and discovery pipelines are drawn out, intricate, and dependent on several variables [116,118,119].

#### Limitations of ML in Late-Stage Drug Development

ML has demonstrated substantial promise in the early phases of drug discovery, particularly in tasks such as target identification, molecular docking, and virtual screening. However, its efficacy diminishes in the later stages of drug development. A key limitation lies in the paucity of data on clinical failures, drug specificity, and long-term toxicity. This data scarcity significantly impairs the predictive performance of ML models.

Notably, the majority of drug candidates that advance to Phase II or Phase III clinical trials ultimately fail, frequently due to unforeseen adverse effects or inadequate therapeutic efficacy in human populations. ML models are constrained by limited access to training data representing these infrequent yet clinically significant outcomes, as only a small fraction of compounds progress to these advanced stages.

This limitation emphasizes the necessity for enhanced data-sharing mechanisms, the incorporation of real-world evidence, and the development of hybrid modeling approaches that integrate mechanistic insights with data-driven ML techniques. Such strategies are essential to improving decision-making processes during the most resource-intensive and uncertain phases of drug development [120].

### 7.4. Natural Language Processing of Medical Records

NLP is used in medical documents. Many medical professionals concur that the drive toward EMR in many countries is slow, complex, and sometimes completely mismanaged. As a result, patients may occasionally receive worse overall care. One of the most significant problems in many hospitals and clinics is the amount of tangible medical data and paperwork. Handwritten notes, inconsistent formatting, and incomplete or dispersed data have hindered EMR implementation.

Since language is the primary medium for conveying complex information, doctors’ notes and annotated medical records include essential information about the population and the health of specific patients. Analysis is difficult due to the language’s unpredictability and inconsistency, and the extraction of higher-level information into relevant subcategories. ML is producing encouraging results when conducting such complex analyses [121].

### 7.5. ML Applications in Prognosis

In clinical practice, prognosis is the process of forecasting the predicted progression of a disease. It also includes determining if symptoms and indicators linked with a given disease will worsen, improve, or remain constant over time, as well as deciding probable associated health issues, complications, capacity to perform everyday tasks, and the possibility of survival. As in a clinical context, multi-modal patient data, such as phenotypic, genomic, proteomic, pathology test findings, and medical pictures, are collected, which may help ML models with illness prognosis, diagnosis, and therapy. ML approaches are employed in various applications, from identifying and categorizing tumors using X-ray and CRT images to detecting cancers using proteomic and genomic (microarray) tests. Scientists have a substantial bias toward using ML to identify outcomes or hazards linked with breast (24%) and prostate (20%) cancer. However, ML technologies seem to effectively forecast results or risks in almost a dozen types of cancer. This shows that machine-learning approaches can be used to forecast and prognosticate cancer in a broad range of situations [122].

The study revealed many similarities in the sorts of ML algorithms employed, the types of training data incorporated, the kinds of outcome predictions made, the types of cancers analysed, and the overall efficacy of these systems in diagnosing cancer risk or outcomes.

### 7.6. ML for Medical Time Analysis

One of the duties of clinical processes is to analyze clinical time-series data. Clinical time-series modeling has been used to assess mean arterial blood pressure (ABP) and intracranial pressure (ICP), two important markers of cerebrovascular autoregulation (CA) in TBI patients, to predict death rates in TBI patients, and to predict intervention strategies in intensive care units (ICUs) using CNN and LSTM. By integrating patient records with multivariate and time-series measurement data, attention models were used in recent studies to track ICU forecasting activities (including diagnosis, estimation, and prediction, among other things) [123].

### 7.7. Prediction of Future Illness Symptoms

Predicting the signs of illnesses that might strike humanity in the future and cause global deaths is vital. ML uses deep neural networks and long-short-term memory learning techniques to forecast the signs and spread of infectious diseases, including malaria, scarlet fever, and chickenpox. Machine-learning algorithms based on information gathered from clinical reports, physician notes, and wearable body sensors may be used to predict patients’ future medical circumstances. The machine learning tool predicts and identifies illness causes based on human genetics.

The project to identify relevant information regarding how genetics can influence human health is currently at the forefront of much scientific knowledge. These are next-generation sequencing (NGS) techniques, the explosion of genetic information, and large databases containing population-scale genetic data. Preventive medicine can benefit from knowing how to picture complex diseases and how an individual’s genetic composition can augment or reduce their risk. This could give doctors more information about how to alter a patient’s course of treatment to minimize the risk of more complicated disorders. The objectives include detecting human activity, tracking health, predicting illness, diagnosing illnesses, and advancing smart medicine. However, the most popular use of machine learning is the development of artificial intelligence infrastructure to make computers smarter, reducing the need for direct patient interaction. Additionally, to enhance current digital technologies so that patients may be recognized, detected, forecasted, and so forth at home. To restrict the worldwide effect of various diseases, machine learning techniques are essential for modernizing our healthcare and other technological systems [123].

## 8. Ethical Considerations in AI-Driven Smart Healthcare

Smart healthcare systems stand to gain significantly from the integration of ML and big data, leveraging these technologies to enhance diagnostic accuracy, personalize treatment pathways, and improve operational efficiency. However, this technological reconfiguration introduces a range of ethical and societal considerations that must be addressed to ensure responsible and equitable implementation.

- Data Privacy and Security: A primary ethical concern involves the privacy and security of patient data. Smart healthcare platforms routinely collect vast volumes of sensitive information from sources such as IoT devices deployed in operating theatres, EHRs, and genomic databases. Safeguarding this data against breaches, misuse, or unauthorized access is not only a legal mandate but also an ethical imperative. In alignment with principles of fair data sharing, emerging technologies, such as blockchain, differential privacy, and secure federated learning, are being increasingly adopted to protect patient data while still enabling its utility for clinical insight generation and ML model development.
- Consent and Transparency: A further ethical challenge arises from issues of consent and transparency. The deployment of black-box ML systems often impedes comprehension of decision-making processes by both patients and clinicians. Explainability must, therefore, be prioritized in the design of ethical AI, particularly in healthcare settings where model outputs may inform critical, high-stakes decisions. Patients should be adequately informed about the use of their data, the risks involved, and their rights regarding contesting or overriding automated decisions.
- Algorithmic Accountability: Equally crucial is the matter of algorithmic accountability. As AI systems become integral to decision-making in diagnosis, treatment planning, and resource allocation, mechanisms for tracing errors, identifying points of failure, and assigning accountability must be clearly established. Health systems must avoid uncritical adoption of these tools in the absence of robust governance frameworks, domain expertise, and risk mitigation strategies to prevent unintended harm.

Beyond these established concerns, deeper ethical dilemmas, often insufficiently addressed, are pivotal for equitable AI integration in healthcare:

- Economic Disparity and Access Limitations: AI-powered healthcare tools often entail high implementation and maintenance costs, disproportionately benefiting wealthier individuals and institutions. This economic imbalance risks widening existing disparities in care quality and health outcomes, particularly in under-resourced settings where financial barriers limit access to advanced diagnostics and AI-supported clinical tools.
- Bias in AI Performance Evaluation: Many AI solutions are developed, validated, and promoted by the same entities, raising concerns regarding inflated performance claims and commercial bias. Clinical robustness should be ensured through independent third-party audits, stringent regulatory oversight, and the use of open, transparent benchmarking methodologies.
- Biases in Historical Healthcare Data: The datasets used to train ML models often reflect historical inequities in healthcare delivery. Consequently, models trained on such data risk perpetuating discriminatory outcomes across race, gender, or socioeconomic status. Ensuring fairness requires rigorous auditing of training datasets, inclusive representation during model design, and continuous model updates to align predictions with equitable health outcomes.

Ultimately, the ethical integration of AI in healthcare necessitates a confluence of technical rigor, transparency, accountability, and inclusivity. This multifaceted approach is essential to the realization of intelligent, trustworthy, and compassionate healthcare systems that serve diverse populations equitably [124,125,126].

## 9. Future Research Direction

Despite the rapid advancement of ML, BDA, and IoMT technologies in healthcare, several critical challenges remain before these revolutions can be safely, effectively, and scalably deployed in real-world clinical environments. Future research must be problem-driven, addressing specific gaps while fostering responsible innovation. This review highlights key trends and limitations within current AI research, identifying privacy and security, scalability and infrastructure, and explainability and clinical integration as dominant themes warranting further scholarly attention.

- Privacy and Security

As the healthcare sector becomes increasingly data-centric, ensuring the secure storage and transmission of sensitive patient information is paramount. Emerging approaches such as locality-sensitive hashing and differential privacy aim to enhance model utility while minimizing direct reliance on identifiable patient data. Future research should prioritize the development of privacy-preserving ML techniques, notably federated learning and differential privacy, which enable distributed model training without compromising data confidentiality. These methods significantly reduce the risk of data breaches while maintaining robust model performance. Moreover, blockchain technology presents a promising avenue for constructing decentralized, tamper-proof, and auditable infrastructures that can facilitate secure and transparent data sharing across institutions and jurisdictions. Such innovations are vital for fostering trust and achieving regulatory compliance in multi-stakeholder healthcare collaborations.

- Scalability and Infrastructure

The exponential increase in health-related data generated by wearable devices, remote monitoring sensors, and EHR systems necessitates the development of scalable and efficient computational infrastructures. ML models must operate with low latency and be capable of delivering real-time analytics in resource-constrained environments, particularly at the network edge. Future research should explore the design of cloud-native and hybrid architectures that can dynamically scale while maintaining data integrity and availability. The establishment of interoperable systems and streamlined data pipelines will be critical to maximizing the utility of big data in smart healthcare ecosystems.

- Explainability and Clinical Integration

The successful implementation of AI in clinical practice hinges on the interpretability of ML models and their ability to provide clinically meaningful explanations. Future work should promote the adoption of explainable AI (XAI) techniques that offer transparent, trustworthy, and actionable insights for clinical end-users. These tools must be rigorously evaluated within real-world healthcare settings to support safe and ethical decision-making. Furthermore, seamless integration into clinical workflows, including embedding AI functionalities within EHR platforms, diagnostic systems, and telemedicine solutions, is essential to ensure usability and clinician acceptance. Research should also focus on the development of standardized evaluation protocols and real-world validation frameworks that assess model robustness, fairness, and generalizability across diverse patient populations.

Addressing these future research priorities is critical to unlocking the transformative potential of ML-powered smart healthcare systems. By ensuring data security, scalable infrastructure, and model transparency, and by enabling clinical usability, future innovations can foster the development of responsible, equitable, and patient-centered AI that meaningfully improves health outcomes across varied clinical settings [127,128].

## 10. Conclusions

Big data and ML in smart healthcare systems offer a transformative opportunity to enhance the efficiency, accuracy, and accessibility of medical treatment. The importance of machine learning techniques in addressing critical healthcare problems such as disease detection, personalized medication, and operational optimization is highlighted in this review. Because BDA and ML work well together, healthcare systems can process massive amounts of data, uncover hidden patterns, and support evidence-based decision-making. Despite these advancements, the industry still confronts significant challenges, such as scalability, interpretability of ML models, and data privacy. The ethical and fair use of these technologies is crucial to establishing broad adoption and building stakeholder trust. Future research should focus on developing robust frameworks for privacy-preserving analytics, enhancing model explainability, and promoting interdisciplinary collaboration to address healthcare data’s unique complexity. By addressing these problems and creating more proactive, patient-centered, and efficient healthcare systems, big data and machine learning have the potential to alter the way healthcare is delivered fundamentally. It will take continual innovation and legislative and regulatory backing to realize these technologies’ potential fully.

Disease prediction, medical imaging, drug development, individualized treatment, EHR management, and remote patient monitoring are just a few ML-driven applications highlighted in the paper. Significant real-world results include:

- Diagnostic accuracy of 95%+ is achieved by ML-integrated platforms, comparable to conventional manual-inspection methods [86].
- Big data-enabled AI diagnostic systems have achieved cost reductions of up to 20%, primarily by minimizing unnecessary testing and streamlining decision workflows [82].
- AI-powered imaging systems developed by NVIDIA and GE HealthCare have been successfully deployed for automated X-ray and ultrasound assessments, improving diagnostic efficiency and expanding access globally [86].
- Oncora Medical is advancing the development of ML algorithms and BDA to establish a standardized and automated solution for oncology treatment planning. The platform aggregates patient-specific clinical data, including tumor genetic profiles, medical histories, imaging results, and prior treatment responses, to assist clinicians in efficiently generating personalized therapy recommendations. This automated approach significantly reduces the interval between diagnosis and the initiation of treatment, thereby enhancing patient outcomes through the provision of timely, precise, and individualized cancer care.
- The UK Biobank uses ML to advance population health research through massive genomic datasets.

These case studies illustrate the tangible advantages of ML and big data in different clinical, research, and operational areas.

Despite apparent progress, the review calls out a range of ongoing challenges:

- Heterogeneity and interoperability of data across systems.
- Privacy and security risks in cloud and IoT-enabled environments.
- Computational hardness in handling high-dimensional, real-time data.
- Lack of black-box ML models’ explainability hinders clinical trust and uptake.
- Scalability issues in scaling ML models across large, distributed healthcare systems.

These constraints impair the seamless integration of intelligent healthcare technologies and require targeted technological and policy interventions.

To facilitate meaningful advancements in the field, future research must provide greater specificity regarding the clinical features under investigation and elucidate the precise mechanisms by which ML and BDA are translated into learning models. It is essential that studies clearly define the patient populations to which predictive algorithms are applicable and distinguish cases where learning models are successfully applied following predictive insight generation from those where no such application occurs.

Subsequent reviews and empirical investigations should adopt a more focused and clinically grounded methodology to reduce redundancy. Specifically, the inclusion of concise, contextually relevant examples, both generalizable and domain-specific, should be prioritized over extensive but superficial commentary. Such an approach is likely to enhance the clarity, accessibility, and practical relevance of ML and big data research for healthcare practitioners, policymakers, and other stakeholders involved in clinical decision-making.

### Limitations of the Review

Although this review offers an extensive overview of the convergence between big data and ML in intelligent healthcare systems, it has several limitations. First, the research is primarily based on a narrative review approach, which, while expansive and exploratory, lacks the statistical sophistication of systematic meta-analyses. Second, literature selection was restricted to English-language publications and potentially excluded relevant non-English language studies. Third, while attempting to address varied applications, some niche use cases and emerging technologies may have been underrepresented due to space and scope limitations. Moreover, the review does not conduct a quantitative comparison of the performance of particular ML models but instead provides conceptual and thematic insights. Lastly, the rapid development of healthcare technologies suggests that newer developments after 2025 may not be included; therefore, ongoing updates are necessary to maintain the findings’ relevance.

## Figures and Tables

**Table 1:** Summary of recent smart healthcare systems research articles.

Focus Area	Proposed Approach	Key Findings	Challenges Addressed	Future Prospects	Study Evaluation	Ref.
Human Activity Recognition (HAR) Using Sensor Data	LSTM-based HAR model Attention and squeeze-and- excitation blocks	99% accuracy in activity recognition; improved feature extraction lower computational complexity	Variability in sensor data; imbalanced Datasets; high computational cost	Enhancing real-world applications of HAR by improving model adaptability and efficiency	Well-defined model; limited to specific dataset; 99% on public HAR dataset	[24]
IoT-based Clinical Decision Support System	Cloud-based C-IoT model with ANN and lightweight encryption for secure health monitoring	91% diagnostic accuracy; health monitoring; enhanced data security	Data security; accuracy	Expanding the model for Society 5.0 by integrating AI-driven secure diagnostics	Small-scale deployment; lacks generalizability; moderate reproducibility	[25]
Intrusion Detection in Smart Healthcare IoT Systems	IADCL framework using feature selection (IRKO), AConBN classifier, and SA-HHO optimization	High accuracy in cyberattack detection using public datasets like CIC-IDS 2017 and 2018	Lack of robust security in resource-constrained IoTM devices	Improving security mechanisms for real-time IoT-based healthcare systems	Benchmark datasets used (CIC-IDS); good methodology; reproducible	[26]
AI-Enabled Smart Acne Diagnosis	Convolutional neural network (CNN)-based cloud-connected IoT device for facial acne severity assessment	AI-driven real-time acne tracking with geographic adaptability, bridging e-healthcare access gaps	Delayed/inaccurate acne diagnosis and lack of remote dermatological consultation	Expanding AI-based dermatology solutions for various skin conditions	Small sample; limited clinical validation; strong conceptual design	[27]
Privacy-Preserving Authentication in IoMT Healthcare	Blockchain-based double anonymity strategy with cross-hospital authentication	Enhanced privacy, decentralization, and a 23–87% reduction in computational costs	Privacy breaches, untraceability, single point of failure in authentication	Implementing blockchain for broader healthcare interoperability	High potential; lacks empirical multi-hospital deployment proof	[28]
Unconstrained Health Monitoring via BCG	Smart wireless flexible sensing system for heart and respiration monitoring	High-sensitivity flexible sensor capturing subtle physiological signals	Discomfort and movement limitations in traditional long-term monitoring	Developing advanced flexible sensors for more wearable health monitoring applications	Strong sensing performance; tested in controlled setup	[29]
Nursing Professional Values and Job Satisfaction	Survey-based analysis using SEM-PLS modeling	Activism and justice are the key influencers of job satisfaction among Vietnamese nurses	Limited professional development and high patient-to-nurse ratios	Studying nursing satisfaction factors across diverse healthcare environments	Statistical rigor; limited to Vietnam; low generalizability	[30]
Secure IoT Communication in Smart Healthcare	Three-factor lightweight mutual authentication scheme with elliptic curve cryptography	Improved security with minimal computational cost, meeting 15 security criteria	Security vulnerabilities in resource-constrained IoT healthcare environments	Enhancing lightweight cryptographic protocols for broader IoT applications	Good cryptographic model; theoretical validation; real-world deployment pending	[31]

**Table 2:** Comprehensive summary of recent research in smart healthcare, IoT security, and emerging technologies.

Focus Area	Proposed Approach	Key Findings	Challenges Addressed	Future Prospects	Study Evaluation	Ref.
Big Data and Gene Therapy in Smart Healthcare	Wearables and tracking devices; early health risk prediction BDA for gene therapy	Early risk identification; before genetic data is available; smart data processing for cost-effective healthcare	Integration of multi-source data; affordability of advanced healthcare analytics	Enhancing predictive healthcare models; expanding data-driven gene therapy applications	Small pilot studies; scalability untested	[37]
Security and Privacy in Smart Healthcare Systems	Comprehensive review of SHS security challenges and proposed solutions	Identifies key risks Cyber threats, privacy issues, and attack vulnerabilities in IoMT	Scalability; complexity; securing interconnected smart healthcare devices	Developing robust security frameworks for safeguarding IoMT-based smart healthcare	Synthesis without empirical validation	[38]
Smart Textiles for Healthcare and IoT Integration	Van der Waals (vdW) force-based 2D functional material integration with textiles	Preserves textile flexibility Adding intelligence; applications (healthcare, human–machine interaction)	Scalability; performance stability Safety concerns in commercial applications	Expansion into fully connected IoT-integrated wearable healthcare systems	Lab prototypes; real-world use pending	[39]
5G Security for IoT and Wearables	Security enhancements in 5G-AKA authentication protocol	Improved user authentication and reduced exposure to security vulnerabilities	Lack of mutual authentication; privacy risks in 5G communication	Developing more secure and resilient authentication mechanisms for next-gen networks	Theoretical analysis; real-world deployment needed	[40]
Self-Powered Wearable Sensors	Triboelectric sensors with AI-based LSTM for sign language recognition	Achieved 96.15% recognition accuracy for sign language patterns	Eliminating external power needs while maintaining accuracy	Expanding self-powered sensors for diverse wearable applications	Limited dataset; controlled conditions	[41]
Secure IoT-based Health Monitoring	Cloud and IoT-based secure health monitoring system	Enhanced security and privacy for wearable healthcare technologies	Data privacy concerns and secure remote patient tracking	Improving security standards for cloud-integrated health monitoring	Limited pilot; reproducibility unclear	[42]
Ensemble Learning for IoT Intrusion Detection	Bagging-based IDS integrating DNN and CNN for IoT security	Improved threat detection using the Edge-IIoTset dataset	Balancing IDS accuracy with computational efficiency	Enhancing real-time IoT security using AI-driven ensemble models	Good on benchmark dataset; edge deployment pending	[43]

**Table 3:** Wearable, home-based, and hospital-based healthcare devices from the perspectives of ML and big data.

Aspect	Wearable Devices	Home-Based Devices	Hospital-Based Devices
Data Collection	Continuous real-time monitoring (e.g., heart rate, steps, SpO_2_)	Periodic monitoring (e.g., BP monitors, glucose meters)	High-frequency; high-resolution medical data (e.g., MRI, CT scans, ECG)
Data Volume	High but fragmented	Moderate to high	Very high (large datasets per patient)
Data Variety	Limited (mainly physiological data)	Moderate (includes environmental and physiological data)	Extensive (medical imaging, lab tests, patient history)
Data Velocity	Real-time streaming	Near real-time to scheduled readings	Batch processing and real-time for critical care
Privacy and Security	Risk of data breaches via cloud or mobile apps	Moderate security concerns (home network vulnerabilities)	High-security standards (hospital IT infrastructure)
ML Applications	Activity recognition; anomaly detection; predictive analytics	Disease monitoring; early warning systems	Diagnosis; precision medicine; treatment planning
ML Model Complexity	Lightweight models (on-device processing)	Moderate complexity (edge/cloud computing)	High complexity (DL AI-driven diagnostics)
Big Data Challenges	Data fragmentation, interoperability issues	Data inconsistency; integration challenges	High computational demands; need for scalable storage
Computational Resources	Low (wearables have limited processing power)	Moderate (some devices leverage cloud/edge computing)	High (dedicated servers, GPUs, Cloud computing)
Integration with Healthcare Systems	Limited (mostly user-driven insights)	Moderate (telemedicine and EHR integration)	Full integration with EHRs and clinical workflows

**Table 4:** Summary of key studies on ML and big data applications in smart healthcare diagnostics.

Study Focus/ Design	Application Domain/ Dataset Size	ML Technique/ Model	Key Contribution/ Reproducibility	Diagnostic Relevance	Ref.
Patient similarity evaluation framework	General healthcare/large-scale patient data	Adaptive semi-supervised recursive tree partitioning (ART)	Efficient patient similarity indexing and retrieval	Supports prognosis, risk assessment, and comparative effectiveness	[61]
AI in operational delivery improvement	Cardiovascular care/healthcare systems	General AI platforms and ML algorithms	Integration of AI in cardiac care operations	Supports diagnosis and risk stratification	[62]
Complaint prediction in diagnostic systems	In-vitro diagnostics/QC data over 90 days	Decision trees, adaptive boosting	Prediction of customer complaints using QC data	Indirect diagnostic support through QC performance monitoring	[63]
Emotion-aware postpartum depression detection	Maternal health/biomedical and sociodemographic data	Ensemble classifiers	Predicts postpartum depression risk	Enables early detection and intervention	[64]
Automated retinal image labeling	Ophthalmology/5000 SEED + public datasets	DL + rule-based classifier	RetiSort for high-accuracy retinal photo sorting	Aids diagnostic preprocessing	[65]
DL in IoT healthcare	Healthcare IoT/44 SLR papers	DL frameworks	Review of DL in healthcare IoT	General healthcare diagnostics	[66]
Fall detection from real-life data	Elderly care/400 days of real-life data	ML models	High-sensitivity fall detection system	Automatic fall detection and alert	[67]
ML in intensive care systems	ICU/general data	ML algorithms	Review of ML clinical decision support	Clinical decision support in ICUs	[68]
Prediction of diabetes complications	Diabetes/147,664 patients	XGBoost	Predicts short and long-term complications	Improves prognosis and care quality	[69]
Non-invasive liver fibrosis diagnosis	Hepatitis C/SLBs data	EMD + ANN-J48	Hybrid intelligent classifier	Non-invasive diagnostic tool	[70]
Federated learning in healthcare	Healthcare/multi-center EHRs	Federated learning	Systematic study of FL in healthcare	Privacy-preserving diagnosis modeling	[71]
Elderly activity tracking	Elderly care/sensor data	HDCO + deep ensemble learning	High-accuracy activity recognition	Behavior monitoring for healthcare	[72]
Predictive maintenance of medical devices	Healthcare equipment/SLR data	ML + big data	Review of predictive maintenance techniques	Ensures device reliability	[73]
IDH prediction in dialysis	Dialysis/patient records	DL	Survey on ML for IDH	Preemptive diagnosis and prevention	[74]
HF phenotyping via AI	Heart failure/VA EHRs (20,000 pts)	NLP + ML (SVM)	Efficient HF identification using EHR	Improved diagnosis in HF registries	[75]
Smart hospital optimization	Hospital care/internal data	NLP + ML + BI	Optimized care, diagnostics, and cost	Enhances diagnostic processes	[76]
AI in rare disease transplant support	Rare diseases/Polish national databases	Big data + AI tools	Policy analysis and recommendations	Supports early diagnosis and treatment	[77]
ML in women’s health	Women’s health/review-based	ML + big data	Perspective on opportunities and biases	Personalized predictive healthcare	[78]
ML for endometriosis detection	Endometriosis/Lucy app (10,000 participants)	Machine learning	Real-world data analysis for earlier detection	Early diagnosis and personalized recommendations	[79]
AI in functional urology	Functional urology/UDS datasets	AI systems	Proposal and discussion on AI use	Improves diagnosis and personalized therapy	[80]

**Table 5:** ML in various healthcare applications and associated data challenges.

Healthcare Application	Role of Machine Learning	Big Data Challenges
Disease Diagnosis	Detects diseases like cancer, diabetes, and neurological disorders using medical imaging and patient data.	Data heterogeneity, interoperability, and privacy concerns.
Predictive Analytics	Forecasts disease outbreaks, patient deterioration, and risk factors.	High-dimensional data processing and real-time analytics.
Medical Imaging	Enhances anomaly detection in X-rays, MRIs, and CT scans using DL.	Large file sizes, data annotation, and storage limitations.
Drug Discovery and Development	Predicts molecular interactions, accelerates drug discovery, and optimizes clinical trials.	Data silos, computational complexity, and regulatory constraints.
Personalized Medicine	Tailors treatments based on genetics, lifestyle, and medical history.	Data integration from diverse sources, ethical concerns.
Remote Patient Monitoring	Analyzes wearable and home-based device data for early intervention.	Real-time processing, connectivity issues, and security risks.
EHR Management	Automates data extraction, summarization, and decision support.	Data inconsistency, duplication, and access control.
Clinical Decision Support	Assists in treatment recommendations using predictive models.	Data accuracy, bias in training data, and model interpretability.
Healthcare Chatbots and Virtual Assistants	Provides symptom checking, appointment scheduling, and medical advice.	NLP limitations, contextual understanding, and data privacy.
Surgical Assistance	Aids robotic surgeries with real-time guidance and precision enhancement.	Sensor data processing and integration with surgical workflows.
Mental Health Analysis	Detects depression, anxiety, and mood disorders using speech and text analysis.	Subjectivity in diagnosis, patient privacy, and data bias.
Fraud Detection and Security	Identifies fraudulent claims and cyber threats in healthcare data.	Anomaly detection in massive datasets, adversarial attacks.

**Table 6:** Comparative analysis of big data and ML applications in smart healthcare systems.

Case Study	Application Area	Technologies Used	Key Outcomes
UK Biobank	Medical Research	BDA ML	Enhanced disease understanding through analysis of extensive health data from 500,000 participants.
NVIDIA and GE HealthCare	Diagnostic Imaging	AI ML	Improved diagnostic accuracy and efficiency in X-ray and ultrasound imaging.
Healthcare Platform	Disease Detection	BDA ML	Achieved over 95% accuracy in disease detection with a 90% cost reduction compared to traditional methods.
Oncology Drug Development	Drug Development	BDA	Streamlined drug discovery processes and personalized cancer treatments.
Oncora Medical	Oncology Workflows	BDAML	Enhanced treatment planning and decision-making in oncology through integrated data analysis.
IQVIA’s NLP Data Factory	Population Health	NLP BDA	Improved population risk stratification by extracting insights from unstructured health data.
Digital Health Platform in Colombia	Population Health Management	BDA ML	Enhanced decision-making and proactive healthcare interventions in resource-constrained settings.
BigQuery ML for Diabetes Prediction, Google Cloud	Diabetes Prediction	BigQuery ML SQL-based ML	Simplified development of predictive models for diabetes risk assessment.
HealthEdge	Type 2 Diabetes Prediction	ML IoT Edge and Cloud Computing	Enabled real-time diabetes risk prediction using integrated IoT-edge-cloud systems.
AI Predicting 10-Year Heart Disease Risk	Cardiovascular Risk Assessment	DL AI	Predicted 10-year risk of heart disease using single chest X-rays, aiding early intervention strategies.

## Data Availability

All data generated or analyzed during this study are included in this published article.
